# Negative impact of anorexia and weight loss during prior pirfenidone administration on subsequent nintedanib treatment in patients with idiopathic pulmonary fibrosis

**DOI:** 10.1186/s12890-019-0841-7

**Published:** 2019-04-11

**Authors:** Satoshi Ikeda, Akimasa Sekine, Tomohisa Baba, Takuma Katano, Erina Tabata, Ryota Shintani, Shinko Sadoyama, Hideaki Yamakawa, Tsuneyuki Oda, Ryo Okuda, Hideya Kitamura, Tae Iwasawa, Tamiko Takemura, Takashi Ogura

**Affiliations:** 1 Department of Respiratory Medicine, Kanagawa Cardiovascular and Respiratory Center, Tomioka-Higashi 6-16-1, Kanazawa-ku, Yokohama, 236-0051 Japan; 2 Department of Radiology, Kanagawa Cardiovascular and Respiratory Center, Yokohama, 236-0051 Japan; 3 Department of Pathology, Japanese Red Cross Medical Center, Tokyo, 150-8935 Japan

**Keywords:** Nintedanib, Pirfenidone, Early termination, Adverse event, Anorexia, Weight loss

## Abstract

**Background:**

Current clinical practice guidelines for idiopathic pulmonary fibrosis (IPF) conditionally recommend use of pirfenidone and nintedanib. However, an optimal treatment sequence has not been established, and the data of treatment sequence from pirfenidone to nintedanib are limited. This study aimed to evaluate safety, tolerability and efficacy of nintedanib switched from pirfenidone in patients with IPF.

**Methods:**

Thirty consecutive IPF cases, which discontinued pirfenidone because of a decline in forced vital capacity (FVC) or intolerable adverse event (AE), and newly started nintedanib (150 mg twice daily) from September 2015 to August 2017 (switch-group) were retrospectively reviewed. Subsequently, we compared the characteristics, treatment status, and AEs between the switch-group and other 64 IPF patients newly started nintedanib during the same period without any prior anti-fibrotic treatment (pirfenidone-naïve group).

**Results:**

In the switch group, median age, body weight, body mass index (BMI), and %FVC were 72 years old, 54.9 kg, 21.0 kg/m^2^, and 52.9%, respectively. Most common AE of nintedanib was aspartate aminotransferase/alanine aminotransferase elevation (71.9%), followed by anorexia (46.7%) and diarrhea (46.7%); whereas, anorexia (63.3%) and ≥ 5% weight loss from baseline (56.7%) were common during pirfenidone administration. Sixteen patients (53.3%) discontinued nintedanib within 6 months (early termination). Multivariate logistic regression analysis revealed a significant association between low BMI and early nintedanib termination in the switch-group (*p* = 0.0239). Nintedanib suppressed FVC decline as compared with that during administration period of pirfenidone in 70% of the patients who could undergo lung function before and after switching to nintedanib. The incidence of early termination of nintedanib was higher in the switch-group than in the pirfenidone-naïve group, whereas body-weight, BMI, absolute FVC values, and %FVC were significantly lower in the switch-group (just before nintedanib initiation) than in the pirfenidone-naïve group. Nintedanib-induced anorexia was more frequent and severer in the switch-group than in the pirfenidone-naïve group, but no significant differences were observed in terms of other AEs.

**Conclusions:**

A high incidence of early termination of nintedanib was noted when patients were switched from pirfenidone. Anorexia and weight loss during prior pirfenidone administration may increase the rate of the early termination of subsequent nintedanib treatment.

## Background

The development of two anti-fibrotic drugs, pirfenidone and nintedanib, has markedly changed the management of idiopathic pulmonary fibrosis (IPF) over the last decade [1, 2]. These drugs have been shown to reduce the decline in forced vital capacity (FVC) among IPF patients with a manageable side-effect profile [3–5] and have received a conditional recommendation for use according to the current clinical practice guidelines of IPF [6]. In addition, nintedanib is expected to restrain the acute exacerbation of IPF; in the INPULSIS® trials, prespecified verification by a central adjudication committee indicated that the risk of acute exacerbation of IPF was significantly lower in the nintedanib group than in the placebo group [4, 7]. A meta-analysis of IPF therapy also revealed that nintedanib, not pirfenidone or NAC, significantly reduced the risk of acute exacerbations development [8]. Moreover, an interim analysis of the INPULSIS®-ON trial indicated that the beneficial effect of nintedanib on slowing disease progression was maintained and the change from baseline FVC was consistent over a long period [9]. On the other hand, pirfenidone has been shown to reduce both all-cause and IPF related mortality in a pre-specified pooled analysis of CAPACITY and ASCEND trials (hazard ratios 0.52 and 0.32, respectively) [10]. Because treatment options for IPF are limited, these two anti-fibrotic drugs should be maximally utilized. However, an optimal treatment sequence has not yet been established. Only one retrospective case series study involving the switching of pirfenidone to nintedanib has been performed [11]. This study indicated that IPF patients switched from pirfenidone due to adverse events (AEs) showed good tolerance of nintedanib, and the intra-individual responses to the two drugs may differ. However, this study had a preliminary nature because of its small sample size (*N* = 7).

The present study evaluated the safety, tolerability, and efficacy of nintedanib in IPF patients switched from pirfenidone to nintedanib for establishing an optimal treatment strategy for IPF in the future.

## Methods

### Patients and settings

This retrospective study was performed at Kanagawa Cardiovascular and Respiratory Center in Yokohama, Japan. All consecutively enrolled patients had (1) IPF diagnosed based on the official American Thoracic Society/European Respiratory Society/Japanese Respiratory Society/Latin American Thoracic Association statement of 2011 [12], (2) discontinued pirfenidone due to a decline in FVC or intolerable AEs despite dose modification or symptomatic therapy, and (3) newly started nintedanib (150 mg twice daily) during September 2015–August 2017. Patients with a history of receiving anti-fibrotic treatments other than pirfenidone or advanced lung cancer complications were excluded. Subsequently, we compared the characteristics, treatment status, and AEs between the aforementioned study participants (switch-group) and other IPF patients who had newly started nintedanib at a dose of 150 mg twice daily during September 2015–August 2017 at our hospital and were without any prior anti-fibrotic treatment or advanced lung cancer complications (pirfenidone-naïve group). This study was performed in accordance with the Declaration of Helsinki. The Ethics Committee of the Kanagawa Cardiovascular and Respiratory Center approved the study protocol (approval date: January 16, 2018; approved number: KCRC-17-0040), and the need for patient consent was waived because this was a retrospective study and anonymity was secured.

### Data availability

The datasets generated and/or analyzed during this study are available from the corresponding authors on reasonable request.

### Clinical and laboratory findings

The clinical and laboratory data were retrieved from patient medical records. Age, gender, height, body weight, laboratory data [aspartate aminotransferase (AST), alanine aminotransferase (ALT), alkaline phosphatase, total bilirubin, γ-glutamyl transpeptidase, serum creatinine, Krebs von den Lungen-6 (KL-6), and Surfactant protein-D (SP-D)], pulmonary function tests, and concomitant therapy were evaluated. Serum levels of KL-6 and SP-D were measured using chemiluminescent enzyme immunoassay system (BML, Inc. Shibuya-ku, Tokyo, Japan).

### Assessment of and response to AEs

The grading of worst examination values of AEs was based on the Common Terminology Criteria for Adverse Events (CTCAE) ver. 4.0 [13]. When a patient developed AEs, treatment interruption or dose reduction was performed in accordance with the guideline for the appropriate use of nintedanib (Ofev®) [14].

### Statistical analysis

Categorical data are presented as numbers (percentages), and compared using Fisher’s exact test. Continuous data are presented as medians (interquartile ranges), and compared using Mann–Whitney U test. A multivariate logistic regression analysis was performed to verify the risk. A *p* value of < 0.05 was considered statistically significant. All statistical analyses were performed using EZR (Saitama Medical Center, Jichi Medical University, Saitama, Japan) [15], which is a graphical user interface for R version 3.2.2 (The R Foundation for Statistical Computing, Vienna, Austria).

## Results

### Characteristics

Thirty IPF patients were enrolled in this study and patient characteristics observed just before initiating nintedanib are summarized in Table 1. Most of the included patients were males (76.5%) and the median age was 72 years old. The median body weight, body mass index (BMI), and body surface area (BSA) estimated using the Du Bois formula were 54.9 kg, 21.0, and 1.59 m^2^, respectively. The median percent predicted FVC (%FVC) and percent predicted diffusing capacity for lung carbon monoxide (%DLco) at baseline were 52.9 and 44.2%, respectively. The physique-related factors, absolute FVC values, and %FVC were considerably lower than those reported in the INPULSIS trials; as for the Japanese patients of nintedanib group in the INPULSIS trials, the mean body weight, BMI, absolute FVC value, and %FVC were 63.8 kg, 24.4, 2.42 L, and 80.9%, respectively. Twenty-seven patients (90%) revealed definite usual interstitial pneumonia pattern on high-resolution computed tomography, and 8 patients (26.7%) have undergone surgical lung biopsy for the diagnosis of IPF.

**Table 1:** Patient characteristics

	Present study (*n* = 30)	Nintedanib group in INPULSIS trials	
		Japanese (*n* = 76)	Overall (*n* = 638)
Baseline characteristics			
Age	72.0 [68.0, 74.8]	68.4 ± 7.6^a^	66.6 ± 8.1^a^
Gender (male/female)	24/6	62/14	507/131
Current or former smoker (%)	26 (86.7%)	66 (86.8%)	464 (72.7%)
Physique			
Body weight (kg)	54.9 [49.7, 64.4]	63.8 ± 11.6^a^	79.2 ± 16.6^a^
Body mass index	21.0 [19.0, 23.6]	24.4 ± 3.4^a^	28.1 ± 4.6^a^
Body surface area (DuBois, m^2^)	1.59 [1.48, 1.72]	–	–
Concomitant therapy			
Prednisolone (%)	3 (10.0%)	9 (11.8%)	136 (21.3%)
Tacrolimus (%)	1 (3.3%)	0	0
Laboratory data			
Aspartate aminotransferase (IU/L)	21.5 [18.0, 24.8]	–	–
Alanine aminotransferase (IU/L)	16.0 [11.0, 24.3]	–	–
Total bilirubin (mg/dL)	0.50 [0.30, 0.60]	–	–
γ-glutamyl transpeptidase (IU/L)	31.0 [21.3, 48.0]	–	–
Creatinine (mg/dL)	0.80 [0.68, 0.87]	–	–
Krebs von den Lungen-6 (U/mL)	1021 [829, 1903]	–	–
Surfactant protein D (ng/dL)	382 [259, 452]	–	–
Lung function test			
Forced vital capacity (L)	1.68 [1.34, 1.99]	2.42 ± 0.67^a^	2.71 ± 0.76^a^
% Forced vital capacity (%)	52.9 [43.7, 69.7]	80.9 ± 16.6^a^	79.7 ± 17.6^a^
% DLco (%)	44.2 [40.9, 58.5]	44.6 ± 11.4^a^	47.4 ± 13.5^a^
Administration history of pirfenidone			
Administration period (months)	8.30 [4.23, 13.9]	–	–
Time from discontinuation to nintedanib initiation			
0 (direct switch)	20 (66.6%)	–	–
< 1 month	3 (10.0%)	–	–
≥ 1 month	7 (23.3%)	–	–
Reason for discontinuation (%)			
Decline of FVC	15 (50.0%)	–	–
Adverse events	15 (50.0%)	–	–
Maintenance dose (%)			
< 1200 mg	10 (33.3%)	–	–
1200 mg	16 (53.3%)	–	–
1800 mg	4 (13.3%)	–	–

Categorical data are presented as numbers (percentages) and continuous data are presented as medians (interquartile ranges)

*Abbreviation*: *DLco* diffusing capacity for lung carbon monoxide

^a^Continuous data in INPULSIS trials are presented as the mean ± standard deviation

Twenty-three patients (76.6%) were switched to nintedanib with an interruption period of less than 1 month, and the median duration of prior pirfenidone treatment was 8.3 months. Fifteen patients (50%) were switched to nintedanib because of FVC decline and 15 patients (50%) because of intolerable AEs. The median follow-up duration from the initiation of nintedanib was 10.4 months (data cutoff date was October 4, 2017).

### Adverse events

The details of AEs during the pirfenidone and nintedanib administration periods are summarized in Table 2. The most common AE of nintedanib was AST / ALT elevation (63.3%), followed by anorexia (46.7%), diarrhea (46.7%), and weight loss (20.0%), whereas the most common CTCAE grade ≥ 2 AE was anorexia (36.7%), followed by diarrhea (26.7%) and AST/ALT elevation (23.3%). By contrast, during the pirfenidone administration period, 19 patients (63.3%) exhibited anorexia, and 16 patients (53.3%) had a CTCAE grade of ≥2. In addition, 17 patients (56.7%) exhibited weight loss with a CTCAE grade of ≥1 (≥5% from baseline), and seven patients (23.3%) had a CTCAE grade of ≥2 (≥10% from baseline).

**Table 2:** Adverse events during the pirfenidone and nintedanib administration periods

	During the pirfenidone administration period (*n* = 30)				During the nintedanib administration period (*n* = 30)			
	Subjects	CTCAE grade			Subjects	CTCAE grade		
		1	2	3		1	2	3
Gastrointestinal								
Anorexia	19 (63.3%)	3	14	2	14 (46.7%)	3	8	3
Weight loss	17 (56.7%)	10	5	2	6 (20.0%)	3	3	0
Diarrhea	0	0	0	0	14 (46.7%)	6	7	1
Dyspepsia	5 (16.7%)	1	4	0	0	0	0	0
Nausea	0	0	0	0	2 (6.7%)	0	2	0
Vomiting	0	0	0	0	2 (6.7%)	2	0	0
Other								
AST/ALT elevation	1 (3.3%)	0	0	1	19 (63.3%)	12	5	2
Fatigue	5 (16.7%)	1	4	0	3 (10.0%)	1	1	1
Photosensitivity	3 (10.0%)	2	0	1	0	0	0	0
Rash	2 (6.7%)	2	0	0	0	0	0	0
Abdominal pain	0	0	0	0	3 (10.0%)	2	1	0
Back pain	2 (6.7%)	0	2	0	0	0	0	0
Non-cardiac chest pain	1 (3.3%)	1	0	0	0	0	0	0
Fever	0	0	0	0	2 (6.7%)	2	0	0
Acute exacerbation	0	0	0	0	2 (6.7%)	0	0	2

Categorical data are presented as numbers (percentages)

*Abbreviations*: *AST* aspartate aminotransferase, *ALT* alanine aminotransferase, *CTCAE* Common Terminology Criteria for Adverse Events

### Treatment status of nintedanib during the observation period

The treatment status during the observation period is summarized in Table 3. Eighteen patients (60.0%) discontinued nintedanib during the observation period. Notably, 16 patients (53.3%) discontinued nintedanib within 6 months. The most common causes of discontinuation were liver injury, anorexia along with weight loss, deterioration of physical condition, death, and acute exacerbation of IPF (10% each). Only 7 patients (23.3%) continued nintedanib without interruption and/or dose reduction.

**Table 3:** Treatment status of nintedanib switched from pirfenidone

	(*n* = 30)
Administration period of nintedanib (months)	5.30 [2.84, 11.8]
Discontinued	18 (60.0%)
Discontinued within 6 months	16 (53.3%)
Reason for discontinuation	
Liver injury	3 (10.0%)
Anorexia + weight loss	3 (10.0%)
Deterioration of physical condition	3 (10.0%)
Death	3 (10.0%)
Acute exacerbation of IPF	3 (10.0%)
Diarrhea	1 (3.3%)
Nausea	1 (3.3%)
Rash	1 (3.3%)
Continued	12 (40.0%)
Continued without interruption/dose reduction	7 (23.3%)
Need at least ≥1 interruption and/or dose reduction	5 (16.7%)
Reason for interruption and/or dose reduction	
Liver injury	2 (6.7%)
Diarrhea	2 (6.7%)
Fever	1 (3.3%)

Categorical data are presented as numbers (percentages)

*Abbreviation*: *IPF* idiopathic pulmonary fibrosis

### Efficacy of nintedanib in patients switched from pirfenidone

**Fig. 1:**
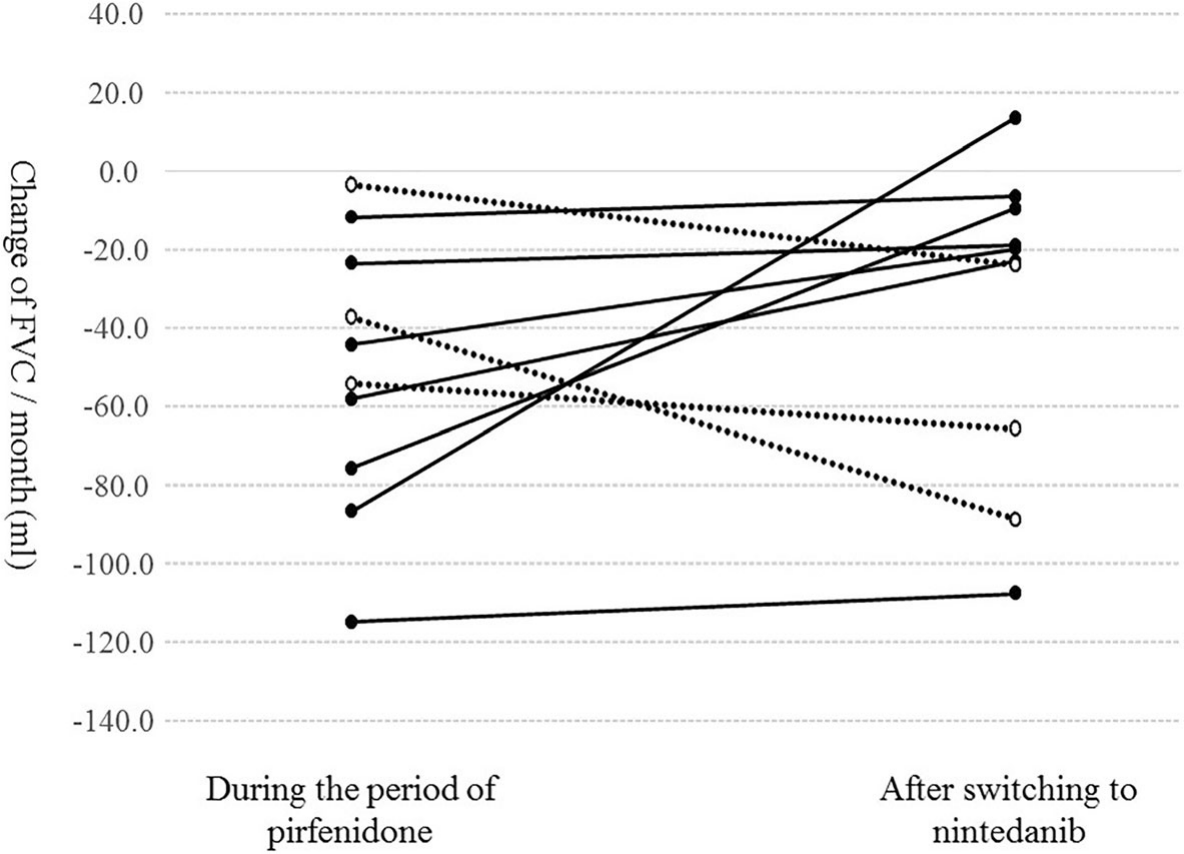
Decline of FVC per month during the administration period of pirfenidone and after switching to nintedanib. Only ten patients could perform lung function tests at 6 months after switching to nintedanib. However, in seven of ten patients (70%), nintedanib suppressed FVC decline compared with that during the administration period of pirfenidone

Figure 1 shows the decline of FVC per month during the periods of pirfenidone administration [(FVC value just before nintedanib initiation – most recent FVC value during pirfenidone administration period) / examination interval] and the decline of FVC per month after switching to nintedanib [(FVC value at 6 months after nintedanib initiation - FVC value just before nintedanib initiation) / examination interval]. Only 10 patients could undergo lung function tests at 6 months after switching to nintedanib. However, in 7 of 10 patients (70%), nintedanib suppressed FVC decline compared with that observed during the pirfenidone administration period.

### Risk factors for the early termination of nintedanib

As stated, 16 patients (53.3%) discontinued nintedanib within 6 months (early termination group), whereas 14 patients (46.7%) received nintedanib for ≥6 months (continuous treatment group). A comparison of the clinical and laboratory data between the two groups (Table 4) revealed that BMI and body weight were significantly lower in the early termination group than in the continuous treatment group (*p* = 0.007 and 0.048, respectively). Logistic regression analysis was performed to verify the risk factors for the early termination of nintedanib (Table 5). Univariate analysis identified a significant association between low BMI and early termination of nintedanib (*p* = 0.0239). We selected BMI not only as the most likely candidate risk factor but also as a representative factor related to physique. We also selected weight loss with a CTCAE grade of ≥2 occurring during the pirfenidone administration period and surfactant protein D (SP-D) (both *P* ≤ 0.1 in univariate analysis) as candidate risk factors. Multivariate logistic regression analysis using backward stepwise selection revealed a statistically significant association between BMI and the early termination of nintedanib (*p* = 0.0111).

**Table 4:** Comparison between the early termination and continuous treatment groups

	Early termination group (*n* = 16)	Continuous treatment group (*n* = 14)	*p* value
Baseline characteristics			
Age	73.0 [67.5, 76.5]	71.5 [68.0, 74.0]	0.601
Gender (male/female)	13/3	11/3	1.00
Physique			
Body weight (kg)	52.6 [47.7, 57.8]	58.5 [54.5, 66.3]	0.048
Body mass index	19.1 [17.2, 21.1]	21.9 [21.0, 24.2]	0.007
Body surface area (DuBois, m^2^)	1.58 [1.47, 1.66]	1.65 [1.51, 1.77]	0.19
Laboratory data			
Creatinine (mg/dL)	0.81 [0.68, 0.87]	0.74 [0.68, 0.86]	0.852
Krebs von den Lungen-6 (U/mL)	1021 [732, 1518]	1047 [845, 2106]	0.678
Surfactant protein D (ng/dL)	315 [186, 393]	420 [273, 553]	0.081
Lung function test			
% Forced vital capacity (%)	48.5 [36.5, 58.6]	56.6 [50.1, 69.7]	0.212
% DLco (%)	45.1 [41.9, 58.6]	42.4 [37.8, 53.3]	0.499
Administration history of pirfenidone			
Administration period (day)	238 [146, 468]	262 [127, 397]	0.934
Reason for discontinuation (%)			
decline of forced vital capacity	6 (37.5%)	9 (64.3%)	0.272
adverse events	10 (62.5%)	5 (35.7%)	
Maintenance dose (%)			
< 1200 mg	8 (50.0%)	2 (14.3%)	0.099
1200 mg	5 (31.2%)	11 (78.6%)	
1800 mg	3 (18.8%)	1 (7.1%)	

Categorical data are presented as numbers (percentages), whereas continuous data are presented as medians (interquartile ranges). Fisher’s exact test was used to compare categorical data, and the Mann–Whitney U test was used to compare continuous data

*Abbreviation*: *DLCO* diffusing capacity for lung carbon monoxide

**Table 5:** Logistic regression analysis for verifying the risk factors for the early termination of nintedanib

	Univariate			Multivariate		
	Odds ratio	95% CI	*p* value	Odds ratio	95% CI	*p* value
Age	1.02	0.885–1.18	0.751			
Gender (male/female)	1.18	0.197–7.08	0.855			
Never smoker (%)	0.857	0.104–7.04	0.886			
Body weight (kg)	0.931	0.861–1.01	0.0702			
Body mass index	0.704	0.519–0.955	0.0239	0.487	0.280–0.849	0.0111
Body surface area (DuBois, m^2^)	0.0418	0.000541–3.22	0.152			
Creatinine (mg/dL)	1.07	0.0280–40.7	0.972			
Krebs von den Lungen-6 (U/mL)	1	0.999–1.00	0.348			
Surfactant protein D (ng/dL)	0.995	0.989–1.00	0.08	0.997	0.990–1.00	0.315
Forced vital capacity (L)	0.561	0.150–2.1	0.391			
% Forced vital capacity (%)	0.967	0.918–1.02	0.213			
% DLco (%)	1.01	0.9580–1.06	0.739			
Administration period of Pirfenidone	1	0.999–1.00	0.329			
Time from pirfenidone discontinuation to nintedanib initiation	1.01	0.994–1.02	0.355			
Discontinued pirfenidone due to FVC decline	0.333	0.0751–1.48	0.148			
Anorexia during the period of pirfenidone	1.65	0.370–7.37	0.512			
Weight loss during the period of pirfenidone	2.93	0.657–13.1	0.159			
Weight loss with grade ≥ 2 during the period of pirfenidone	7.8	0.804–75.6	0.0764	3.29	0.184–58.8	0.418

Univariate analysis showed that the association between low body mass index and the early termination of nintedanib was statistically significant (*p* = 0.0239). In addition, the association between weight loss with a CTCAE grade of ≥2 during the pirfenidone administration period and the early termination of nintedanib was also marginally significant (*p* = 0.0764). A multivariate logistic regression analysis using backward stepwise selection showed that the association between BMI and the early termination of nintedanib was statistically significant (*p* = 0.0111)

*Abbreviation*: *DLCO* diffusing capacity for lung carbon monoxide

### Comparison between the switch and pirfenidone-naïve groups

A comparison of the baseline characteristics (observed just before nintedanib initiation) and data related to nintedanib therapy between the aforementioned study participants (switch-group, *N* = 30) and other IPF patients who were newly started on nintedanib without any prior anti-fibrotic treatment at our hospital (pirfenidone-naïve group, *N* = 64) revealed that body weight, BMI, %FVC, and %DLco were significantly lower in the switch-group than in the pirfenidone-naïve group (Table 6). Conversely, the incidence of nintedanib-induced anorexia was significantly higher in the switch-group than in the pirfenidone-naïve group (*p* = 0.028). Moreover, nintedanib-induced anorexia tended to be more severe in the switch-group than in the pirfenidone-naïve group (Table 7). The proportion of discontinuation of nintedanib within 6 months was also higher in the switch-group than in the pirfenidone-naïve group, although this difference did not reach statistical significance (*p* = 0.0720). However, just before initiating pirfenidone, patients in the switch-group had approximately the same body weight, BMI, BSA, FVC, and DLco values as the baseline values of the pirfenidone-naïve group.

**Table 6:** Comparison between the switch and pirfenidone-naïve groups

	Switch-group (*n* = 30)		Pirfenidone-naïve group (*n* = 64)	*p* value
	Just before initiation of pirfenidone	Just before initiation of nintedanib		
Characteristics				
Age	71.0 [67.0, 75.0]	72.0 [68.0, 74.8]	72.0 [65.8, 75.3]	0.903*
Gender (male/female)	24 / 6	24 / 6	53 / 11	0.778*
Physique				
Height (cm)	165 [158, 169]	165 [158, 169]	164 [160, 170]	0.958*
Body weight (kg)	61.4 [59.2, 66.0]	54.9 [49.7, 64.4]	63.2 [54.2, 73.1]	0.01*
Body mass index	22.6 [21.0, 24.9]	21.0 [19.0, 23.6]	23.9 [20.7, 26.2]	0.001*
Body surface area (DuBois, m^2^)	1.69 [1.62, 1.76]	1.59 [1.48, 1.72]	1.68 [1.54, 1.82]	0.063*
Lung function test				
Forced vital capacity (L)	2.36 [1.78, 3.52]	1.68 [1.34, 1.99]	2.21 [1.74, 2.66]	0.001*
% Forced vital capacity (%)	62.5 [51.0, 76.6]	52.9 [43.7, 69.7]	67.7 [55.9, 79.0]	0.001*
% DLco (%)	58.4 [46.7, 65.7]	44.2 [40.9, 58.5]	54.8 [47.6, 67.9]	0.009*
Nintedanib				
Administration period (month)	–	5.30 [2.84, 11.8]	6.13 [3.02, 14.5]	0.415
Discontinue within 6 months	–	16 (53.3%)	21 (32.8%)	0.072
Adverse events				
AST/ALT elevation	–	19 (63.3%)	46 (71.9%)	0.475
Diarrhea	–	14 (46.7%)	34 (53.1%)	0.659
Anorexia	–	14 (46.7%)	14 (21.9%)	0.028
Weight loss	–	6 (20.0%)	6 (9.3%)	0.188
Nausea	–	2 (6.7%)	11 (17.1%)	0.213
Fatigue	–	3 (10.0%)	5 (7.8%)	0.707

Categorical data are presented as numbers (percentages), whereas continuous data are presented as medians (interquartile ranges). *p values were calculated by comparing the baseline characteristics of the switch-group just before nintedanib initiation and the baseline characteristics of the pirfenidone-naïve group. Fisher’s exact test was used to compare categorical data, and the Mann–Whitney U test was used to compare continuous data

*Abbreviations*: *DLCO* diffusing capacity for lung carbon monoxide, *AST* aspartate aminotransferase, *ALT* alanine aminotransferase

**Table 7:** Details of adverse events of nintedanib in the switch and pirfenidone-naïve groups

	Switch-group (*n* = 30)				Pirfenidone-naïve group (*n* = 64)			
	Subjects	CTCAE grade			Subjects	CTCAE grade		
		1	2	3		1	2	3
Gastrointestinal								
Anorexia	14 (46.7%)	3	8	3	14 (21.9%)	8	2	4
Diarrhea	14 (46.7%)	6	7	1	34 (53.1%)	20	10	4
Weight loss	6 (20.0%)	3	3	0	6 (9.3%)	2	3	1
Nausea	2 (6.7%)	0	2	0	11 (17.1%)	5	5	1
Vomiting	2 (6.7%)	2	0	0	3 (4.7%)	3	0	0
Dyspepsia	0	0	0	0	1 (1.6%)	0	1	0
Other								
AST/ALT elevation	19 (63.3%)	12	5	2	46 (71.9%)	28	12	6
Fatigue	3 (10.0%)	1	1	1	5 (7.8%)	2	3	0
Abdominal pain	3 (10.0%)	2	1	0	3 (4.7%)	3	0	0
Fever	2 (6.7%)	2	0	0	4 (6.3%)	4	0	0
Acute exacerbation	2 (6.7%)	–	–	–	3 (4.7%)	–	–	–
Pneumothorax	0	–	–	–	3 (4.7%)	–	–	–
Rash	0	0	0	0	2 (3.1%)	0	2	0
Hemoptysis	0	–	–	–	1 (1.6%)	–	–	–
Cerebral infarction	0	–	–	–	1 (1.6%)	–	–	–
Eosinophilia	0	–	–	–	1 (1.6%)	–	–	–
Pneumatosis intestinalis	0	–	–	–	1 (1.6%)	–	–	–
Hematochezia	0	–	–	–	1 (1.6%)	–	–	–
Thrombocytopenia	0	–	–	–	1 (1.6%)	1	0	0

Categorical data are presented as numbers (percentages)

*Abbreviations*: *AST* aspartate aminotransferase, *ALT* alanine aminotransferase, *CTCAE* Common Terminology Criteria for Adverse Events

## Discussion

In the present study, as many as 53.3% of patients discontinued nintedanib within 6 months after switching from pirfenidone. Although risk factors for the early termination of nintedanib in IPF patients have not been fully investigated, the present study demonstrated the following two important clinical observations; first, low BMI was a risk factor for the early termination of nintedanib in the switch group; second, nintedanib-induced anorexia was more frequent and severer in the switch-group than in the pirfenidone-naïve group, but no significant differences were observed in terms of other AEs.

The present study suggested that a small physique can predict the early termination of nintedanib. In fact, the incidence of early termination of nintedanib in the present study was considerably higher than that in the INPULSIS trials, whereas values of physique-related factors, such as body weight, BMI, and absolute FVC, were considerably lower than those reported in the INPULSIS trials. Similarly, in the present study, the incidence of early termination of nintedanib was higher in the switch-group than in the pirfenidone-naïve group, whereas the values of physique-related factors were significantly lower in the switch-group than in the pirfenidone-naïve group. Interestingly, just before initiating pirfenidone, patients in the switch group had approximately the same body weight, BMI, BSA, and absolute FVC values as the baseline values of the pirfenidone-naïve group. This down-sizing of physique in the present study might have been due to not only the disease progression but also pirfenidone-induced weight loss, and this would be a major problem in the treatment sequence from pirfenidone to nintedanib.

Furthermore, the high incidence of early termination in patients with a small physique may have been because of the increase in the incidence and severity of AEs. We previously reported that a high incidence of hepatotoxicity resulting in treatment interruption was noted in IPF patients treated with nintedanib at our hospital [16]. In this study, small physique was associated with the hepatotoxicity of nintedanib in IPF patients. Similarly, despite the relatively short observation period of the present study, the incidence and severity of nintedanib-induced AEs, such as AST/ALT elevation and anorexia, tended to be higher in this study than in the INPULSIS trials. A pharmacokinetic study confirmed that body weight is a statistically significant covariate that influences nintedanib exposure [17]. Based on these results, we speculated that small patients tended to have a high serum concentration, and were therefore more likely to develop AEs. Careful monitoring of AEs and dose adjustment of nintedanib is required especially for the small patients.

However, although the values of physique-related factors were significantly lower in the switch-group than in the pirfenidone-naïve group in the present study, only nintedanib-induced anorexia was significantly more frequent and severer in the switch-group than in the pirfenidone-naïve group, whereas no significant differences were observed in other AEs such as diarrhea and AST/ALT elevation. During the pirfenidone administration period before being switched to nintedanib, 53.3% of patients exhibited anorexia with a CTCAE grade of ≥2, and 56.7% of patients exhibited weight loss of ≥5% from baseline. Nevertheless, 76.6% of patients were switched to nintedanib with an interruption period of less than 1 month. The anorexia and weight loss occurring during the pirfenidone administration period persist until nintedanib initiation and might affect the incidence and severity of anorexia during the subsequent nintedanib treatment. In fact, among 14 patients who developed anorexia during the nintedanib administration period in the switch group, 11 patients (78.6%) have already experienced anorexia during prior pirfenidone treatment. Furthermore, in the switch group of the present study, anorexia along with weight loss was one of the most common immediate causes of nintedanib discontinuation (Table 3), and this might also be the underlying cause of deterioration in physical condition or death. With regard to body weight loss, this could be an independent factor for decreased survival of IPF [18]. Thus, careful monitoring of body weight and the maintenance of nutritional status is mandatory in patients receiving anti-fibrotic therapies.

It is also noteworthy that nintedanib suppressed FVC decline compared with pirfenidone in 70% of patients who could undergo lung function tests before and after switching to nintedanib. As indicated both in a previous case series [7] and the present study, the intra-individual response to the two anti-fibrotic drugs may differ, and nintedanib is expected to suppress disease progression even after deterioration during the pirfenidone administration period. However, our results indicated that pirfenidone administration continued until the appearance of anorexia with a CTCAE grade of ≥2 or weight loss is inappropriate to maximally utilize subsequent nintedanib. Considering that gastrointestinal AEs tended to occur early in the treatment course (< 6 months) of pirfenidone [19], dose modification or symptomatic therapy is required during this period [20]. When the gastrointestinal AEs of pirfenidone cannot be adequately managed, clinicians should consider switching to nintedanib early, although continued treatment with pirfenidone may suppress further FVC decline and/or death even in patients with IPF who exhibit meaningful disease progression during treatment [21]. Moreover, when clinicians consider switching from pirfenidone to nintedanib due to gastrointestinal AEs, it would probably be better to have a certain washout period.

Recently, several clinical trials of the combined use of pirfenidone and nintedanib, which mainly evaluated the safety and pharmacokinetics, have been reported [22–24]. To proficiently use two anti-fibrotic drugs, there is a need to accumulate more cases and conduct further research into combination therapy or a treatment sequence involving nintedanib followed by pirfenidone administration.

A limitation of the present study was the retrospective single-center study design. Additionally, the number of included patients was small and the distribution of patients may have been skewed. There is a need to accumulate more cases from several hospitals and conduct further investigations for the validation of the present results. The short observation period was also a limitation when assessing long-term safety. Moreover, we didn’t set specific criteria for the FVC decline to consider switching from pirfenidone to nintedanib in our center, thus leaving the attending physicians to determine whether pirfenidone treatment is to be continued or changed.

## Conclusions

A high incidence of early termination of nintedanib was noted when patients were switched from pirfenidone. Anorexia and weight loss during prior pirfenidone treatment may increase the rate of early termination of subsequent nintedanib treatment. Further investigation is required to establish an optimal treatment strategy.

## Abbreviations

AE: Adverse event
ALT: Alanine aminotransferase
AST: Aspartate aminotransferase
BMI: Body mass index
BSA: Body surface area
CTCAE: Common Terminology Criteria for Adverse Events
DLco: Diffusing capacity for lung carbon monoxide
FVC: Forced vital capacity
IPF: Idiopathic pulmonary fibrosis
KL-6: Krebs von den Lungen-6
SP-D: Surfactant protein D
